# Temporal Feature Perception in Cochlear Implant Users

**DOI:** 10.1371/journal.pone.0045375

**Published:** 2012-09-21

**Authors:** Lydia Timm, Deepashri Agrawal, Filipa C. Viola, Pascale Sandmann, Stefan Debener, Andreas Büchner, Reinhard Dengler, Matthias Wittfoth

**Affiliations:** 1 Department of Neurology, Hannover Medical School, Hannover, Germany; 2 Department of Psychology, Neuropsychology Lab, Carl von Ossietzky University of Oldenburg, Germany; 3 Department of Otorhinolaryngology, Hannover Medical School, Hannover, Germany; 4 NeuroImaging and Clinical Applications (NICA), Hannover, Germany; University of Salamanca- Institute for Neuroscience of Castille and Leon and Medical School, Spain

## Abstract

For the perception of timbre of a musical instrument, the attack time is known to hold crucial information. The first 50 to 150 ms of sound onset reflect the excitation mechanism, which generates the sound. Since auditory processing and music perception in particular are known to be hampered in cochlear implant (CI) users, we conducted an electroencephalography (EEG) study with an oddball paradigm to evaluate the processing of small differences in musical sound onset. The first 60 ms of a cornet sound were manipulated in order to examine whether these differences are detected by CI users and normal-hearing controls (NH controls), as revealed by auditory evoked potentials (AEPs). Our analysis focused on the N1 as an exogenous component known to reflect physical stimuli properties as well as on the P2 and the Mismatch Negativity (MMN). Our results revealed different N1 latencies as well as P2 amplitudes and latencies for the onset manipulations in both groups. An MMN could be elicited only in the NH control group. Together with additional findings that suggest an impact of musical training on CI users’ AEPs, our findings support the view that impaired timbre perception in CI users is at partly due to altered sound onset feature detection.

## Introduction

A cochlear implant (CI) can restore hearing in humans with severe and profound sensori-neural hearing loss. While bypassing the outer and middle ear, acoustical signals are converted into electric pulses and directly stimulate the inner ear, e.g., the hearing nerve fibers. Although electrical activation of auditory pathway through a CI differs fundamentally from natural hearing, most CI users are able to interpret sound percepts as meaningful and can derive information enabling them to successfully understand speech. Since the CI was mainly created as a prosthesis to enhance speech perception, music perception remains in comparison poor [1], [2], with outcomes depending on the complexity of the musical stimuli. Successful perception of rhythm and - to some extent - of pitch can usually be obtained, while the fine structure of sound information is still missing leading to unpleasant music sensations [3], [4], [5].

Timbre is defined as ‘that attribute of sound by which a listener can judge that two sounds having the same loudness and pitch are dissimilar’ [6]. A sound’s timbre is depending on the spectral and temporal envelope, it is developing and its perception is crucial for musical appraisal and practical tasks such as instrument identification. CI users face the problem of impaired instrument identification through which auditory scene analysis is particularly impaired in complex music environments such as orchestral concerts or band music [7], [8]. While most studies have investigated the spectral cues needed for a sufficient timbre and music perception [9], [10] only a few focused on the temporal envelope of sound [11], [12]. Kong and colleagues used a multi dimensional scaling paradigm in which participants had to rate similarities between instruments. Their findings suggested that CI users made their judgements according to the temporal envelope cues of sounds, and relied on attack time information [13]. Heng and colleagues corroborated these finding using auditory chimeras built from one instruments spectral and another instruments temporal envelope and found that CI users tend to judge the instrument mainly in regard to the temporal envelope information [12].

Three major elements can be described in the temporal evolvement of a sound: (i) the attack time in which the spectral information is still under transition, (ii) the sustain time in which the tone/sound enfolds its whole spectrum and reached a steady state, and (iii) the decay time in which the sound vanishes. The first element depends on the excitation mechanism of the instrument and varies in the time domain from instrument to instrument. If the attack time is manipulated, instrument identification and differentiation is less successful and error rates increase as shown in psychoacoustical studies by Berger and Iverson [14], [15]. If the temporal envelope with its fine structure is providing the most crucial information and is not well implemented in the CI’s processing strategy, the CI user has a low chance of detecting differences in the attack time of different instruments. However, the variance in performance of music perception in CI users is large, suggesting that additional factors such as musical training (pre- and post-implantation), practicing of a musical instrument, and the frequency of listening to music may influence the outcome of music related tasks, like pitch and instrument differentiation [16].

In contrast to standard tests of music perception abilities with behavioral measurements, the use of auditory event-related potentials (AEPs) has the advantage of not relying on subjective impressions. AEPs have been successfully used as an objective method assessing musical sound perception in CI patients [17], [18]. In contrast, functional neuroimaging techniques such as positron emission tomography (PET) and functional magnetic resonance imaging (MRI) appear less feasible to study brain functioning in CI users because of the invasive characteristic and safety concerns, respectively [19].

Though the literature on AEPs in CI users is still limited, several studies point to the usability of AEPs as a biomarker for perceptual changes and brain plasticity [20], [21]. Accordingly, Tremblay and colleagues have suggested that the N1–P2 complex could be used to monitor neurophysiologic changes during auditory training for CI users [22], [23]. Furthermore, the mismatch negativity (MMN), an AEP component elicited by infrequent auditory stimuli deviating from regular standard sounds, has emerged as a reliable marker for CI users’ ability to accurately discriminate stimuli without the trade-off of subjective behavioral responses [17], [24], [25], [26]. A mismatch negativity (MMN) is known to indicate the detection of differences between the sensory memory trace of a preceding stimuli and the present one [27]. In addition, it is largely attention-independent and sensitive to small changes in stimulus features near to the just notable difference thresholds [28].

Sandmann and colleagues successfully used an MMN paradigm developed by Naatanen and colleagues showing that MMNs may be elicited in CI users [17], [29], [30]. Furthermore, Stoody and colleagues presented a successful MMN paradigm for CI users when exposed to spectral modulations [31]. Accordingly in our study we employed an oddball paradigm in which mismatch negativity-like deflections in response to rare deviant sounds can be expected in CI users and NH controls [28].

We used a cornet sound, which was manipulated in the temporal domain. Sounds were presented either with a natural unchanged temporal envelope, a sound envelope in which the attack time was shortened by 60 ms, or with an envelope with artificially prolonged attack time (prolonged by 60 ms). Previous AEP studies on NH controls have shown that the encoding of the physical attributes of sounds, including the detection of stimulus onset, are reflected within the N1–P2 complex [32]. Weise and colleagues have demonstrated how the transition between the different parts of the temporal envelope is decoded and how this decoding is represented in N1 and P2 amplitudes and latencies [33], [34]. We therefore expected that in our AEPs manipulations of attack time should be reflected in N1–P2 latencies and amplitudes, reflecting the encoding of the physical properties of the stimuli as well as feature detection and onset deviation, respectively To further investigate whether the encoding of temporal sound features in CI users was appropriately represented in auditory sensory memory [34], we compared the MMN between NH controls and CI users for all deviant conditions. We anticipated that the MMN in both groups would be found for all conditions and would show group specific differences in amplitudes and latencies.

## Materials and Methods

### Behavioral Testing

In order to evaluate the discriminability of the stimuli, an additional group of CI users with congruent demographics as those taking part in the EEG study (e.g. Freiburger Monosyllabic words test in quiet, duration of deafness, see Table S1) underwent a behavioral discrimination task in a pilot study. CI users (N = 12) (age range in years: 22–56, mean age: 37.4, SD: 12.4) and an age-matched NH control (N = 12) group (age range in years: 28–53, mean age: 36.5, SD: 11.78) had to distinguish between the three manipulated attack times of the Horn sound. Stimuli were presented in sets of three, where two stimuli were identical while one was a deviant. The position of the deviant was altered within the task to prevent order effects. Each deviant occurred 20 times resulting in 40 trials. We applied this three-alternative choice task as it is a procedure often used in clinical context and known to most CI users. Participants were asked to indicate the position of the deviant by pressing on a corresponding key on a keyboard.

### Participants EEG Study

Fifteen CI users and fifteen age- and sex-matched normal-hearing individuals (NH controls) participated in the study. The AEP N1 signal-to-noise ratio (SNR), which was estimated as the root mean square of the N1 peak at Fz divided by the root mean square of the baseline (−100 to 0 ms) in dB, was used as an exclusion criterion at 10 dB. Since no clear AEPs could be observed in three of the CI users (SNR <10 dB), these participants were excluded from further analyses. To keep the two groups equal, the exclusion of the three CI users led to the exclusion of their match from the NH group, resulting in 12 CI users (age range in years: 22–59, mean 44.6; SD 10.5) and 12 NH controls (age range in years: 26–55, mean 45.3; SD 7.8) in the final analysis (see Table 1 for detailed patient characteristics). Prior to the experiment, all CI users had been using their implant for at least 12 months. Additionally, their hearing abilities exceeded 20% in the Freiburger monosyllabic words test in quiet environment, a standard speech intelligibility test in which participants repeat monosyllabic words presented at a level of 65 dB. The correct answers are calculated in percentage.

**Table 1:** Demographical data of CI users of the EEG study.

Subject	Sex	Age(years)	Implanted side	Implant type	Dur. Of deafness(years)	Dur. of Implantuse (month)	Musical training	Speechscore Freiburgermonosyllabic words testin quiet (%)
P1	f	48	right	Nucleus	6.33	184	yes	85
P2	f	59	right	Nucleus	1.91	95	yes	50
P3	f	42	left	Nucleus	2.67	32	yes	95
P4	f	43	right	ABClarion	1.34	176	yes	85
P5	f	49	left	Nucleus	1.08	13	yes	85
P6	m	37	Right	MedelSonata	0.61	21	yes	65
P7	m	56	left	AB Hires	2.92	19	no	70
P8	m	22	left	Nucleus	8.17	41	no	25
P9	f	42	right	Nucleus	2.34	20	no	65
P10	f	57	left	AB Hires	6.26	39	no	90
P11	m	47	left	Nucleus	3.76	20	no	65
P12	f	34	right	AB Clarion	1.59	96	no	90

Participants of the EEG study filled out a modified musical questionnaire, the Ollen Musical Sophistication Index (OMSI) which asked about their extent of musical experience and musical training [35]. Furthermore, this questionnaire included a Likert scale of music appreciation (1 =  dislike listening to music, 7 =  enjoy listening to music very much) and asked about daily/weekly musical practise.

### Ethics Statement

All procedures were approved by the ethics committee of Hannover Medical School and the study protocol conformed to the declaration of Helsinki. Participants gave written informed consent before data collection.

### Stimuli

**Figure 1:**
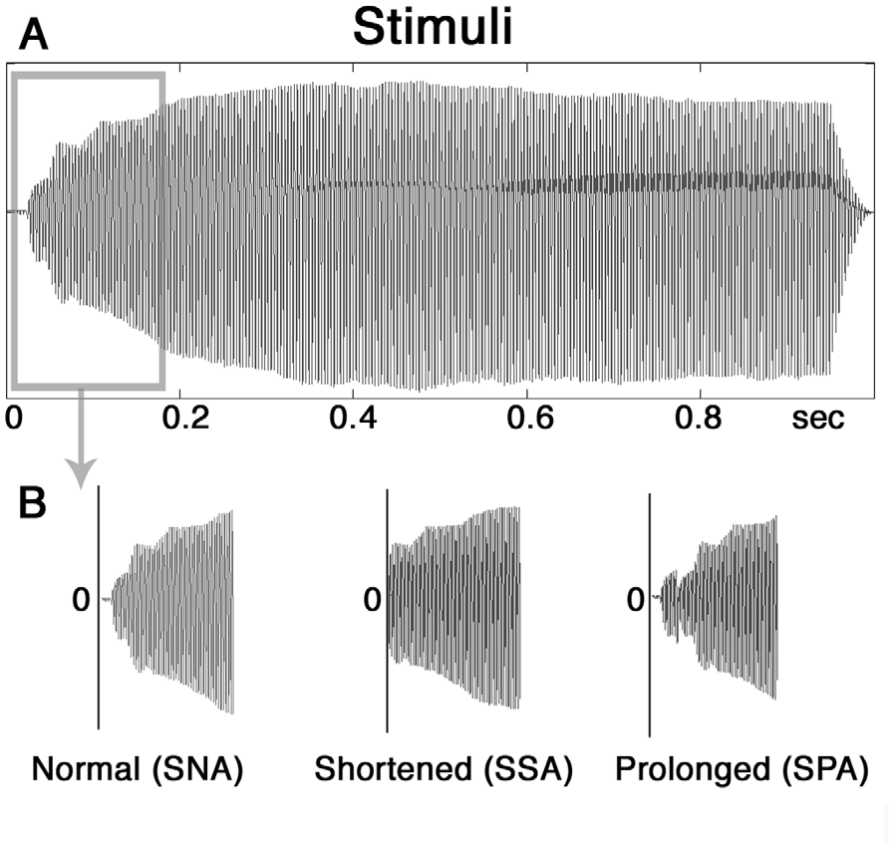
Waveform of experimental stimuli. A: Waveform of the overall stimuli; B: Different attack time manipulations of the stimuli.

All participants listened to a sampled (360 Hz) French horn sound. The stimulus was taken from the ultimate-sound-bank-ircam-solo-instruments (www.ircam.fr, 24-bit/44.1kHz resolution) and manipulated in Pro Tools 9 (www.avid.com/de/products/family/pro-tools). Stimulus duration was kept at 900 ms for all stimuli. The stimuli were presented in three different incarnations. First, we used a non-manipulated sound (NA) as given by the aforementioned database. For the second condition the sound was manipulated by cutting off the first 60 ms yielding a sound with shortened attack time (SA) carried out with a standard digital audio workstation by removing the respective samples. For the last condition the attack time was prolonged (PA). Thus, the samples cut off for the (SA) condition were added at the beginning of the sound. To achieve a smooth transition, a crossfade was employed between the two elements of the sound (see Figure 1). The three different stimuli (NA, SA, PA) were presented in an oddball paradigm with one of them as the frequent standard (SNA/SSA/SPA) tone and the other two as deviants (DNA/DSA/DPA). The probability of deviant sound presentation was 20%. To account for order effects, three blocks were built consisting of 360 stimuli each. The standard sound of the first block became the deviant of the next, whereas one of the former deviants became the standard sound in the second block and so on. To prevent order biases, blocks were split in two parts (A_1_A_2_; B_1_B_2_; C_1_C_2_) and presented in different orders across participants. Inter stimulus interval was one second, resulting in a total experimental duration of 34. 2 minutes ((360×3 stimuli) × 1900 ms = 34.2 min). Participants were seated in a sound-attenuated and electrically-shielded room in front of a monitor and watched a silent black and white movie during stimulus presentation (passive listening). For both groups loudness was individually adjusted to a moderate level which is equivalent to 60–70 dB [36]. For both groups, the intensity of the presented sounds reached therefore approximately 65 dB.

Stimuli were presented via two loudspeakers placed left and right beneath the monitor.

### EEG Recordings

EEG was recorded with a BrainAmp system from 30 scalp channels using active electrodes (Acticap, Brain Products, Munich, Germany) placed according to the 10–20 system [37]. A unipolar montage was used to record the electro-oculogram: one electrode was placed at the outer canthus of the right eye and the second below the right eye. The nose-tip was used as common reference for all channels. Sampling rate was 250 Hz, the data were analogue filtered from 0.1 to 80 Hz, and electrode impedances were kept below 10 kΩ. For the CI users, three to six channels mainly from the temporal (T12/T8) to the occipital electrodes (P08) had to be unattached due to interferences with the implant transmission coil (channels range: 3–6, mean: 3, SD: 1).

### Data Processing

EEG data were analyzed in MATLAB (Mathworks, Nattick, MA, USA) environment using EEGLAB 9.0.5.6b [38]. The data were filtered offline using a FIR filter with the lower edge of the frequency pass band at 1 Hz and a higher edge of the frequency pass band at 30 Hz. Data were screened for extreme values exceeding −200 to 200 µV, as well as for infrequent and un-stereotyped artifacts using the inbuilt probability function (pop_jointprob) with a threshold of 3 SD [39]. For further artifact attenuation, Infomax independent component analysis (ICA) was applied. Ocular and cardiac artifacts were identified using the CORRMAP plug-in [40], and ICs found to reflect blinks, lateral eye movement and cardiac artifacts were removed from the data in CI users and NH controls (removed ICs mean in CI users: 4.8; SD: 4.8, NH controls: 5; SD: 1.2). Since the usage of a cochlear implant causes electrical interference with the EEG recording [39], [41], [42] further ICs reflecting CI artifacts were identified by visual inspection. Evaluation of IC topography centroid (on the implant side) and time course enabled artifact identification as reflecting CI artifact and were based on the same ICA as ocular and cardiac removal (removed CI ICs mean: 2.4; SD: 1.2).

Following ICA-based artifact attenuation, data were segmented into epochs from −100 to 500 ms relative to stimulus onset, since both the N1–P2 complex and the MMN are known to be elicited typically in this time range. For the CI group, the missing channels were spherically interpolated with respect to the neighboring channels. Single subject averages were computed for each participant using a baseline from −100 to 0 ms. To quantify the quality of AEPs, signal-to-noise ratios (SNRs) were calculated for the stimulus NA in both groups (n = 30) based on a single subject level at electrode Fz. SNRs were obtained by dividing the absolute of the N1 peak amplitude by the root mean square of the baseline (−100 to 0 ms) in a time window of 100 −180 ms. AEPs were further analyzed only for those participants who had SNRs above 10 dB (N = 12), as well as peak morphologies discernible as N1 [43]. All statistical analyses were carried out on a fronto-central electrode (Fz), which is known to show strong N1 and MMN responses [27]. In order to quantify differences in the N1 latency range, we selected a time window from 100 to 200 ms after stimulus onset for the unaltered and prolonged sounds (SNA, SPA) and a time window from 80 to 180 ms for the shortened sounds. For optimal latency measurement, a jackknife approach [44] was applied, as this method has been shown to be robust against outliers and single subject variability. In this approach, for all participants in each group (n = 12) grand averages of n–1 are computed, leading to 12 grand averages per group, each consisting of 12 subsamples from which latency measures were extracted.

Analysis of the N1 peak amplitude was carried out in a time window (120–180 ms) defined by the results of the latency jackknife procedure. To avoid biases from noisy data or double peaks, the N1 peak amplitude was calculated as an average of time points surrounding the maximum, resulting in a peak average time window of 32 ms. For further evaluation, we conducted a visual inspection of the N1 topographies in the respective time range.

For analyses of the P2 latency, the jackknife procedure was applied in a time window of 180–240 ms. Similar to the analysis of the N1 AEPs, the P2 peak amplitude in these time windows was calculated as a mean in a 32 ms window around the peak.

The oddball paradigm allowed us to compute difference waves evaluating MMNs in three different conditions. As opposed to the traditional deviant minus standard subtraction, we evaluated MMNs for the identical sound, occurring once as a deviant in one block and once as standard in another block. This procedure prevents any bias due the physical properties of different sounds and allows more direct comparison of ERPs [45].

The time windows for MMN extraction for CI users and NH controls were based on a sample by sample t-test computation, which indicated significant differences between the AEPs of standard and deviant condition. The sample by sample t test computation revealed for the CI users, a time window of 190 to 240 ms (NH controls:120 to 168 ms) for the condition DNA-SNA (no attack manipulation), 152 to 200 ms (NH controls:132 to 180 ms) for the condition DSA-SSA (shortened attack), and 204 to 252 ms (NH controls: 172 to 220 ms) for the condition DPA-SPA (prolonged attack) after stimulus onset. To examine amplitudes we calculated the mean of the difference waves in the respective time windows.

### Statistics

All statistics were carried out in SPSS 19. For behavioral data we examined stimuli in dependency on occurring position and deviation with prolonged or 60 ms shortened attack). A general linear model with repeated measure ANOVA (group*condition*position) was computed to evaluate the hit rates depending on the deviant category (within-subjects), the position (within-subjects), and the group (between-subjects). To assess group specific and stimulus specific differences we applied post hoc t-tests when appropriate.

N1 and P2 component latencies and amplitudes were compared in 4 general linear models (GLM) with repeated measure ANOVA with condition as within subject factor (SNA, SSA, SPA) and Group as between-subjects factor (CI user vs. NH controls). Post hoc t-tests were carried out to examine stimulus and group dependent differences.

Additional statistical analyses with the same GLMs were applied on two subgroups (n = 6) of CI users. The division was according on the OMSI results and divided the CI users group into a musically trained and musically untrained group based on self-reported musical exercise/training (actively singing in a choir or playing an instrument) of at least 15 minutes a day, or 2 hours a week post implantation.

In order to investigate whether MMN amplitudes differed significantly from zero, one–sample t-tests were conducted. Group specific differences were tested with a repeated measures ANOVA with MMN condition as within-subjects factor and Group as between- subjects factor. This analysis was also applied to the CI users subgroup.

For all statistical analysis of the latency measures, the t and F values were corrected as t = t/n–1 and F =  F/(n–1)^2^, as needed in jackknifing method [44].

Greenhouse Geisser correction was applied when necessary and will be reported with unchanged degrees of freedom and epsilon.

## Results

### Behavioral Data

**Figure 2:**
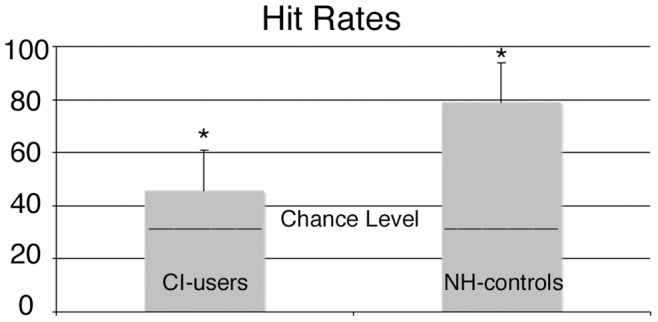
Hit rates of CI users and NH controls *(Asterisks indicate a significant above chance level p<0.05).*

As indicated in Fig. 2 participants of the discrimination task in the pilot study showed hit rates above chance levels in both groups (CI mean = 45.83% SD = 6.3; p = 0.03; NH mean 79.25%; SD = 5.8; p = 0.001, chance level: 33%) as tested with chi-squared test.

For the ANOVA (group*condition*position) we found an interaction for stimulus and position (F_2,44_ = 21.02; p<0.001, ε = .989), a three way interaction with stimulus, position and group (F_2,44_ = 3.23; p = 0.05, ε = .989) as well as a group effect with F_1,22_ = 14.31; p = 0.001 (ε = .989). Group specific differences were tested with post hoc t-test and showed significantly better performance for NH controls for the deviant with shortened attack when presented on second or third position (2^nd position^: t = −3.85, p = .001, 3^rd position^: t = −2.94, p = .008) and the deviant with the prolonged attack presented on 3^rd^ position(t = −4.96, p<.001). Deviants presented on the first position did not show group specific differences (DSA 1^st^ p = .23, DPA 1^st^ :p = .29).

In the CI users group post hoc paired t-tests showed significant lowest hit rate for DSA when presented on the third position compared to DSA when presented on first position (t = 2.60, p = .025) or second position (t = 2.32, p = 0.04). For DPA CI users scored significantly higher when presented on first position compared to presentation on third position (t = 2.63, p = .023).

For the NH controls we found significant differences only for deviant DSA. When presented on second position, NH controls showed highest hit rate compared to presentation on first (t =  −4.3, p = .001) or third position (see figure S1 for further details).

CI users showed no significant difference in their overall hit rates for DPA and DSA, when hit rates were analyzed independent of the stimulus position (p = .12), neither did NH controls show a significant difference for DPA and DSA (p = .87).

Independent samples t-tests showed no differences between the two groups of CI users (behavioral and electrophysiological paradigms) for different individual factors, including age (p = .698), speech perception ability (Freiburger monosyllabic words test in quiet: p = .157), and duration of deafness (p = .055) (see table S1 for detailed information).

Music scores from the Ollen Musical Sophistication Index (OMSI) as used for the EEG paradigm showed no significant differences between CI users and NH controls in the extent of musical experience and musical appreciation ((scaling 1–7) CI mean = 5.18, SD = 1.47; NH mean = 6.30; SD = 1.26; p>0.5). Findings revealed six musically trained subjects in the CI users group (>15 minutes daily training), whereas four musically trained subjects were in the NH control group. None of the participant had received professional practice lessons or underwent any music courses at a university. No significant differences were observed for the appreciation of listening to music between the CI users (mean: 5.1; SD: 1.4) compared to the NH controls (mean: 6.4; SD: 1.4; p = 0.064).

### EEG

The AEPs from CI users showed only low residual CI artifacts and confirmed the validity of the ICA approach in this context [39]. We examined the quality of the N1 peak by means of SNR with a mean of 15.59 dB (SD: 4.29) for CI users and a mean of 21.35 dB (SD: 6.38) for NH controls. A one-way ANOVA showed significant differences (F_1,23_ = 6.70; p = 0.017) between CI users and NH controls.

**Figure 3:**
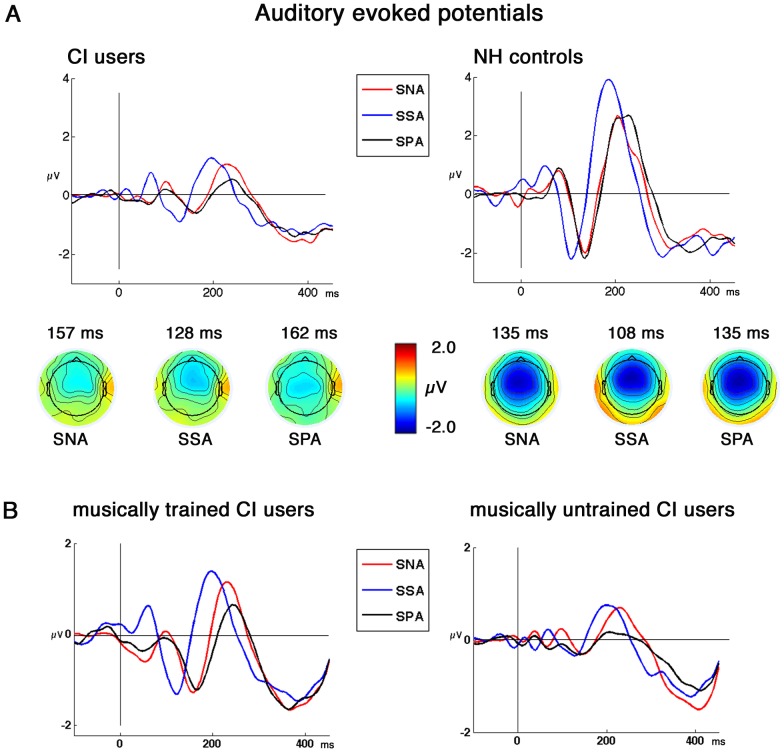
Grand average AEPs of CI users and NH controls. A: Grand average AEPs of CI users and NH controls with topographies of the N1 for the three standard sounds with their corresponding latencies (SNA: Standard Normal Attack; SSA: Standard Shortened Attack, SPA: Standard Prolonged Attack). B: Grand average AEPs of CI users with (N = 6) and without (N = 6) musical training. All grand averages are deflections at Fz.

For the N1 latencies, a repeated measures ANOVA showed a significant within-subjects effect (F_(2,44)_ = 8.61; p<0.001, ε = .75). As illustrated in Fig. 3, the N1 peak latency was modulated by the stimuli. For the CI users, SSA lead to the shortest latency, differing significantly from SNA (t = 5.79, p<.001) and SPA (t = 3.96, p<.001) as shown by post hoc paired t-test. For NH controls the shortest latency was likely obtained for SSA, differing significantly from SNA (t = 15.3, p>.001) and SPA (t = 7.54, p<.001). We found no significant difference between condition SNA and SPA in either group (CI users: t = 0.63, p>0.1; NH controls: t = 0.12. p>0.1). The repeated measures ANOVA on the N1 latencies revealed also a significant between-subjects effect (F_(1,22)_ = 3.86; p<0.05), and post hoc t-tests showed significantly delayed N1 latencies for CI users when compared to NH listeners. (SNA: t = −2.4, p<.05; SSA: t =  −2.7, p<.001; SPA: t = 2.64, p>.001) (for detailed latencies values see Table 2). Similarly, N1 amplitudes were significantly different between the two groups as revealed by a significant between-subjects effect (F_1,22_ = 10.56; p = 0.004). Post hoc independent t-tests revealed significantly smaller N1 amplitudes in CI users than in NH controls for SNA (t = −3.1, p = .005), SSA (t = −2.3, p = .02) and SPA (t = −3.1, p<.001). For the repeated measures ANOVA on N1 amplitudes, however, we did not observe a significant within-subjects effect (p = .67) or an interaction (p = .56).

**Table 2:** AEP latencies (ms) and amplitudes (µV) of CI users and NH controls (SD given in parentheses).

	CI users				NH control		
AEP	SNA	SSA	SPA		SNA	SSA	SPA
N1 latency	157 (±1)	128 (±2)	162 (±2)		135 (±1)	107 (±1)	135 (±2)
P2 latency	236 (±2)	197 (±2)	242 (±3)		204 (±2)	186 (±2)−2.2 (±1)	231 (±2)
N1 amplitude	−0.6 (±1)	−1.0 (±1)	−0.5±(0.7)		−2.1 (±1)		−2.1 (±1)
P2 amplitude	1.2 (±1)	1.3 (±0.9)	0.7 (±1)		2.4 (±1)	3.8 (±1)	2.8 (±0.9)
	musically trained CI users				musically untrained CI users		
N1 latency	155 (±3)	126 (±2)		166 (±2)	134 (±16)	133 (±2)	138 (±6)
P2 latency	230 (±4)	196 (±3)		248 (±3)	223 (±6)	194 (±3)	200 (±3)
N1 amplitude	−1.3 (±0.6)	−1.7 (±0.7)		−0.8 (±0.3)	−0.5 (±0.9)	−0.6 (±0.8)	−0.7 (±0.7)
P2 amplitude	1.3 (±1)	1.5 (±1)		0.9 (±1)	0.6 (±0.2)	0.5 (±0.8)	.05 (±0.1)

The P2 latencies showed a within-subjects effect (F_(2, 44)_ = 3.75; p<0.05,ε = .76), following the pattern of the N1 latencies, with SSA exposing the shortest P2 latency compared to SNA (CI users: t = 2.18, p<.05; NH controls: 186 ms) and SPA (CI users: t = 5.69, p<.001; NH controls: ). We did not observe a between-subjects effect for the P2 latencies (p>0.1).

For the P2 amplitudes, we observed a significant within-subjects effect (F_2,44_ = 8.78; p = 0.001) and a significant between-subjects effect (F_1,22_ = 18.16; p<0.001) as well as an interaction (F_2,44_ = 4.07; p = 0.024). CI users’ P2 amplitudes to SNA was most prominent and differed significantly when compared to SPA (t = 2.38, p = .036), whereas SNA showed no significant difference when compared to SSA (t = .53, p = .60). Significant difference was also obtained for P2 amplitude between SSA and SPA (t = 2.46, p = .031) with SSA exposing larger amplitude. In the NH control group significant differences were found for P2 amplitudes of SSA when compared to SNA (t = 4.06, p = .002) and SPA(t = 3.3, p = .007). No significant P2 amplitude differences where observed for comparison of SNA and SPA (t = .81, p = .43) for the NH controls. The groups differed significantly from each other for P2 amplitude of SSA (t = 5.4, p<.001) and SPA (t = 4.8, p<.001) with larger amplitudes obtained for NH controls, but not for SNA (t = 1.9, p = .068).

ANOVAs for N1 amplitudes in the CI group with condition as within-subjects factor and musical training vs. no musical training as between-subjects factor revealed larger amplitudes for musically trained CI users as indicated by a significant within-subjects effect (F_2,20_ = 3.58; p = 0.047) and a significant between-subjects effect (F_1,10_ = 8.51; p = 0.015). Amplitudes differed significantly between the two groups for condition SNA (t = −2.47, p = .03) and SSA (t = −3.06, p = .01) with larger amplitudes exhibited in the musical trained CI users group. No group specific difference was found for SPA (t = −2.15, p = .057).

For the N1 latencies we found a significant within-subjects effect (F_1,10_ = 3.98, p<.05) but no between-subjects effect (p = .41).

For P2 amplitudes we observed no within-subjects effect (p = .07) or between-subjects effect (p = .614). We also did not find any significant effect for P2 latencies (within-subjects: p<0.1; between-subjects: p = .40).

Six one-sample t-test were carried out on the difference waves, indicating two significant MMN amplitudes for the condition DNA-SNA (t: −2.28, p = 0.043) and condition DPA-SPA (t: −2.68, p = 0.021), specifically in NH controls. For the CI users group no significant MMN amplitudes were observed (see table 3). While repeated measures ANOVA with Condition (3)*Group showed no within-group (p = .20) or interaction effects (p = .46), we found a significant between-subjects effect for MMN amplitudes (F_1,22_ = 4.67; p = 0.42). For MMN latencies we found no within-subjects (p>0.1) or between-subjects effects (p>0.1).

**Table 3:** MMN latencies and amplitudes of CI users and NH controls.

		CI users					NH controls			
Parameters	mean(µV)	SD	t	p	latency(ms)	mean(µV)	SD	t	p	latency(ms)
MMN_DNA-SNA_	−.35	1.1	−1.09	.29	233	−.74	1.1	−2.28*	.043	164
MMN_DSA-SSA_	−.02	1.2	−.06	.95	170	−.17	1.1	−0.52	.607	161
MMN_DPA-SPA_	−.07	1.1	−.21	.83	251	−.83	1.0	−2.68*	.021	221

*The asterisks indicates the level of significant threshold.*

* *p<0.05.*

**Figure 4:**
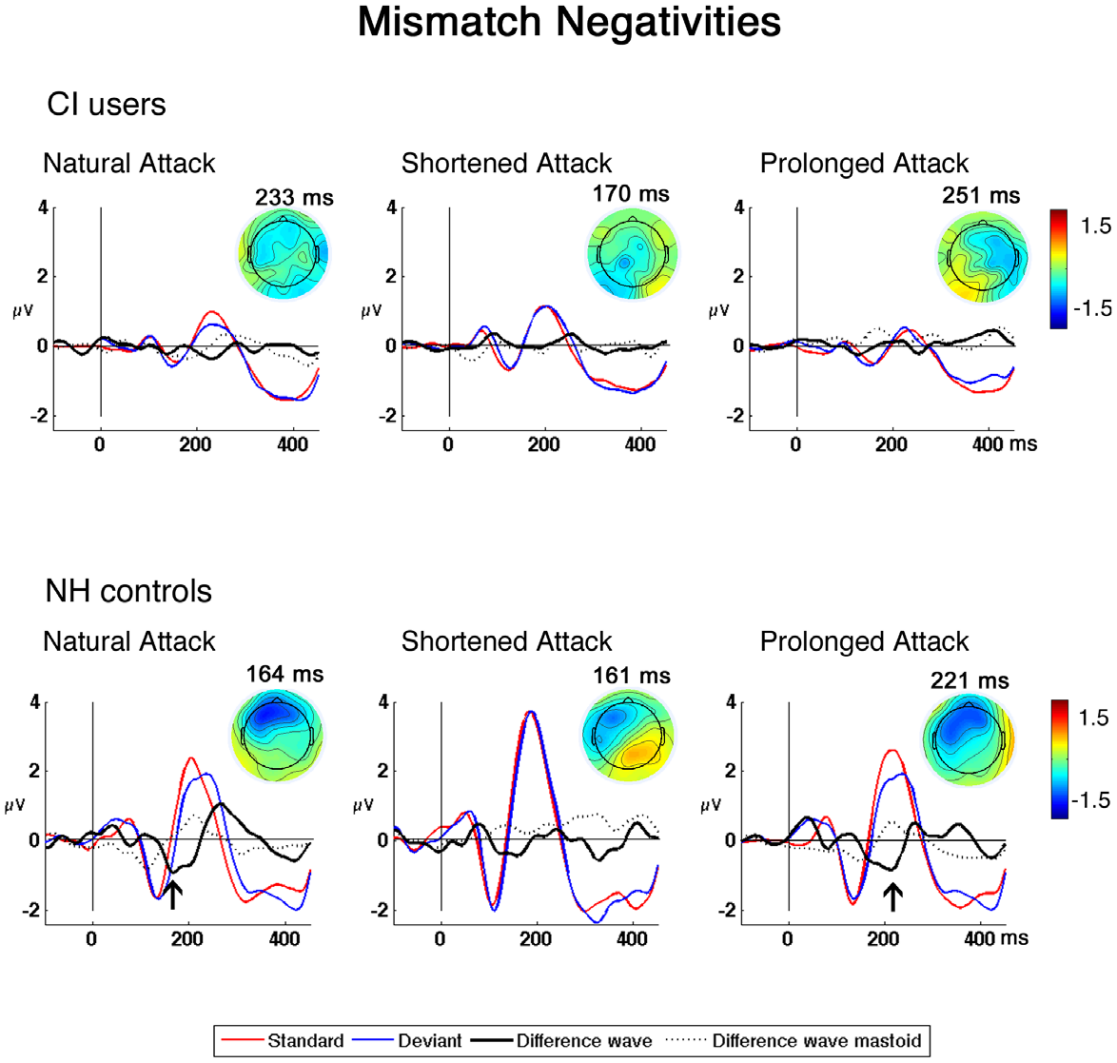
Grand average Mismatch Negativities. Grand average MMNs of CI users and NH controls with their respective topographies. AEPs at Fz for standards (red) and deviants (blue) for CI users and NH controls. Significant MMNs in the difference wave (black) are marked with an arrow. The difference waves for reversed polarity were derived from the left mastoid (dotted black line).

Figure 4 shows that the two CI user subgroups did not reveal any significant MMNs, and the GLM did not show a within-subjects (p = .80) or a between-subjects effect (p = .31), neither for amplitudes nor for latencies (within-subject: p>0.3; between-subjects: p = 1.2).

## Discussion

In the present study, our aim was to investigate abilities in discriminating the temporal envelope of sounds, in particular the attack time in postlingually deafened CI users. We used sets of stimuli that were manipulated in the first 60 ms of the sound. Even though our electrophysiological and behavioral measurements were conducted on two different groups of CI users, it is likely that the different stimuli were detectable and discriminable for CI users and NH controls above chance level. However, while ICA enabled recovery of AEPs in CI users, low residual electrical artifacts were present in the data. Due to the limited number of channels used in the present study we could not eliminate all of the artifacts driven by the implant, as the ICA algorithm works best when supplied with a high number of scalp sensors [46]. Nevertheless, the time window corresponding to the auditory responses of interest was free of artifacts.

### Behavioral Data

CI users were able to distinguish stimuli from each other, whether they showed a prolonged, shortened, or natural onset. The differences between CI users and NH controls were largest for the overall hit rate and showed no specific differences for deviant category. Our results for the behavioral paradigm are congruent with earlier studies showing less accuracy in CI users than in NH controls when confronted with complex stimuli, especially in timbre related tasks [5], [47], [48]. However, CI users showed a hit rate above chance, indicating the possibility of detecting the aforementioned differences in attack time. This corroborates the findings by Kong and colleagues [13], [49], indicating that for timbre judgement CI users mainly rely on the temporal envelope which is perceived as dominant cue for instrument differentiation.

### N1–P2 Differences between CI Users and NH Listeners

Temporal envelope manipulation elicited different N1 characteristics in NH controls and CI users. In both groups, the latency range of the N1 significantly decreased when attack time was shortened. These findings show that CI users and NH controls were able to process the differences in the manipulated stimuli. However, there was a large difference in amplitude between CI users and NH controls but not between the different conditions within the groups.

These differences were driven by the steepness of the temporal envelope and therefore due to different intensities at earlier or later time points of the stimuli depending on the manipulations. Studies on intensity functions of AEPs in CI users and NH controls demonstrated decreased N1–P2 latency and increased amplitude with increased stimulus intensity [50]. We did not observe amplitude changes for different stimulus types but only latency differences in the N1. These findings lead to the conclusion that the N1 latencies are representing rather changes in the temporal domain than only an intensity change and might, for example, be driven by onset encoding of the stimulus material [34]. Since the N1 is particularly sensitive to the spectrotemporal features at the onset of an auditory stimulus, we are confident that the observed N1 differences represent attack time manipulations of the sound [51].

### N1–P2 in CI Users with and without Musical Training

Of special interest is the outcome of the CI users undergoing musical training. In our study the musical trained CI users reported their training following the CI surgery.

This self reported musical training in terms of instrument practicing resulted in more prominent N1 peaks in the musically trained CI users when compared to the N1 amplitudes of the untrained CI users. Even though the different CI users in our subgroup reported heterogeneous learning instruments, their N1 amplitudes rather approach those of the NH control group level, which indicates an effect of musical training on AEP developing. These effects might be discussed from two perspectives. Either the musical training itself is contributing to the development of the normalized AEPs in CI users, or the more frequent exposure to musical stimuli resulting from the training might foster the effect.

The benefit of musical training for auditory rehabilitation in CI users has been discussed before, as several studies have shown improved performance of CI users in music related tasks after structured musical training [3], [48], [52], [53], [54]. Furthermore, the differences between the two groups were only found for the N1, but not for the P2 component, suggesting that musical training specifically has an impact on sound feature detection. Experience-related enhancement of N1 and P2 responses has been shown in healthy participants before. Shahin and colleagues demonstrated how latencies and amplitudes are influenced in non-musicians and musicians undergoing musical training [55].

With regard to CI users, however, the effect of musical training has been evident only from behavioral studies [53]. Here, we show that musical training after implantation seems to be reflected in AEPs as well. Further studies with larger sample sizes of musically trained and untrained participants are necessary to foster AEP-driven findings.

### MMN

The mismatch negativity is thought to reflect an automatic process, which detects a difference between a new stimulus and the preceding stimuli of a sensory memory trace [28], [34], [56]. Driven from our behavioral findings we expected the MMN to be observed in NH controls and likely in CI users. Although, CI users showed differences in the N1 latency between different conditions, these participants failed to produce a significant MMN in any condition. NH controls on the other hand showed a robust MMN for the first condition in which the unaltered sound as a standard (SNA) was subtracted from the unaltered sound occurring as a deviant (DNA) and the third condition, in which the prolonged standard (SPA) was subtracted from the prolonged deviant (DPA). As MMN amplitudes are in general highly correlating with behavioral discrimination thresholds [57], the lack of significant MMNs in CI users might be caused by the narrow physical difference between the standards and deviants, even though behavioral data indicated discrimination above chance-level. However, while a condition effect was found for the N1 latency, the small physical difference between our stimuli seem not salient enough to facilitate auditory sensory memory encoding. The total absence of MMN in CI users was unexpected since a recent study by Heng and colleagues, using auditory chimaeras has shown that CI users orient their musical perception more on temporal envelopes than on fine structure of the spectrum [12]. One might speculate whether our results differ because of the small difference in attack times, since Heng and colleagues manipulated all elements of the temporal envelopes including amplitudes. A second explanation might be the behavioral hit rate, which already showed less accuracy in CI users than in NH controls. While the subjects underwent the behavioural task in an alert state and were fully concentrating on the physical differences between the stimuli, the subjects performed a passive listening task in the MMN paradigm. Thus, the non-significant MMNs in the CI users group might be driven by the limited saliency the stimuli evoked, as seen in earlier studies with a discrepancy between behavioral and MMN results [58].

The differences we have observed in CI users for the N1 but not for the MMN for all conditions may be explained by the theory of basic sensory analysis [59], [60]. An auditory stimulus is analyzed by two parallel channels. One is the N1-generating “transient detector system” which registers sudden energy changes in the auditory environment (e.g. the on and offset of a sound). The second channel is the “feature-detector-system” which analyzes auditory stimuli’s physical features, such as frequency, intensity and duration. Accordingly, our stimuli lead in both groups to a sufficient decoding of the transient detector system and thus modulated N1 responses, while it was not sufficiently excited in the CI user group and thus failed to produce an MMN.

Although no significant MMNs were elicited in CI users, we encourage further research in this field, as the general ability of CI users’ deviance detection mechanism was successfully shown in studies in which the oddball paradigm was exchanged by a new fast paradigm [17]. In this paradigm several deviant categories, such as pitch and intensity are presented in a different probability compared to the oddball paradigm, leading to a shorter experimental duration [29], [30]. Nevertheless, CI users failed to produce MMNs to certain deviant categories in this paradigm. Sandmann and colleagues showed that although MMNs were elicited for frequency and intensity deviations, duration deviants did not elicit any significant MMNs, although behavioural performance was highly above chance level. In line with other studies on sound discrimination abilities in CI users, we therefore conclude that small acoustic differences are difficult to perceive for CI users and further research is necessary to examine the minimal and maximal borders of this altered perception.

### Conclusions

Our findings of N1 and P2 latency differences corroborate the current literature on the use of AEPs as an objective measure in CI research [18], [39], [41]. Here, we showed that even small sound differences might be appropriate to assess the general hearing abilities of CI users. We speculate that the lack of significant MMNs reflects the impairment of CI users to properly integrate deviating onsets, therefore explaining altered musical sound perception, in particular the perception of timbre. We therefore encourage musical training in CI users since it has been shown to strongly affect AEPs of CI users.

## Supporting Information

Figure S1 Hitrates for manipulated attack time Deviants per Position. Hitrates for different attack time manipulations in dependency on their position for CI users (Group1) and NH controls (Group 2). Asterisks indicate level of significance *p<0.05; **p<0.001.
(TIF)

Table S1 Demography of the behavioral discrimination task participants.
(DOC)

## References

[1] Cooper WB, Tobey E, Loizou PC (2008) Music perception by cochlear implant and normal hearing listeners as measured by the Montreal Battery for Evaluation of Amusia. Ear Hear 29: 618–626. 18469714 10.1097/AUD.0b013e318174e787 PMC2676841

[2] Koelsch S, Wittfoth M, Wolf A, Muller J, Hahne A (2004) Music perception in cochlear implant users: an event-related potential study. Clin Neurophysiol 115: 966–972. 15003780 10.1016/j.clinph.2003.11.032

[3] Leal MC, Shin YJ, Laborde ML, Calmels MN, Verges S, et al (2003) Music perception in adult cochlear implant recipients. Acta Otolaryngol 123: 826–835. 14575398 10.1080/00016480310000386

[4] Gfeller K, Oleson J, Knutson JF, Breheny P, Driscoll V, et al (2008) Multivariate predictors of music perception and appraisal by adult cochlear implant users. J Am Acad Audiol 19: 120–134. 18669126 10.3766/jaaa.19.2.3 PMC2677551

[5] Limb CJ, Rubinstein JT (2012) Current research on music perception in cochlear implant users. Otolaryngol Clin North Am 45: 129–140. 22115686 10.1016/j.otc.2011.08.021

[6] ANSI (1973) American national standard psychoacoustical terminology. New York: American National Standard Institute.

[7] McDermott HJ (2004) Music perception with cochlear implants: a review. Trends Amplif 8: 49–82. 15497033 10.1177/108471380400800203 PMC4111359

[8] Galvin JJ 3rd, Fu QJ, Oba SI (2009) Effect of a competing instrument on melodic contour identification by cochlear implant users. J Acoust Soc Am 125: EL98–103. 19275282 10.1121/1.3062148 PMC2677289

[9] Drennan WR, Rubinstein JT (2008) Music perception in cochlear implant users and its relationship with psychophysical capabilities. J Rehabil Res Dev 45: 779–789. 18816426 10.1682/jrrd.2007.08.0118 PMC2628814

[10] Won JH, Drennan WR, Kang RS, Rubinstein JT (2010) Psychoacoustic abilities associated with music perception in cochlear implant users. Ear Hear 31: 796–805. 20595901 10.1097/AUD.0b013e3181e8b7bd PMC2965810

[11] Pressnitzer D, Bestel J, Fraysse B (2005) Music to electric ears: pitch and timbre perception by cochlear implant patients. Ann N Y Acad Sci 1060: 343–345. 16597784 10.1196/annals.1360.050

[12] Heng J, Cantarero G, Elhilali M, Limb CJ (2011) Impaired perception of temporal fine structure and musical timbre in cochlear implant users. Hear Res 280: 192–200. 21664263 10.1016/j.heares.2011.05.017 PMC3343076

[13] Kong YY, Mullangi A, Marozeau J, Epstein M (2011) Temporal and spectral cues for musical timbre perception in electric hearing. J Speech Lang Hear Res 54: 981–994. 21060140 10.1044/1092-4388(2010/10-0196) PMC3107380

[14] Iverson P, Krumhansl CL (1993) Isolating the dynamic attributes of musical timbre. J Acoust Soc Am 94: 2595–2603. 8270737 10.1121/1.407371

[15] Berger KW (1964) Some factors in the recognition of timbre. J Acoust Soc Am 36: 1888–1891.

[16] Gfeller K, Olszewski C, Rychener M, Sena K, Knutson JF, et al (2005) Recognition of “real-world” musical excerpts by cochlear implant recipients and normal-hearing adults. Ear Hear 26: 237–250. 15937406 10.1097/00003446-200506000-00001

[17] Sandmann P, Kegel A, Eichele T, Dillier N, Lai W, et al (2010) Neurophysiological evidence of impaired musical sound perception in cochlear-implant users. Clin Neurophysiol 121: 2070–2082. 20570555 10.1016/j.clinph.2010.04.032

[18] Sandmann P, Eichele T, Buechler M, Debener S, Jancke L, et al (2009) Evaluation of evoked potentials to dyadic tones after cochlear implantation. Brain 132: 1967–1979. 19293240 10.1093/brain/awp034

[19] Giraud AL, Truy E, Frackowiak R (2001) Imaging plasticity in cochlear implant patients. Audiol Neurootol 6: 381–393. 11847465 10.1159/000046847

[20] Sharma A, Dorman MF (2006) Central auditory development in children with cochlear implants: clinical implications. Adv Otorhinolaryngol 64: 66–88. 16891837 10.1159/000094646

[21] Kraus N, Micco AG, Koch DB, McGee T, Carrell T, et al (1993) The mismatch negativity cortical evoked potential elicited by speech in cochlear-implant users. Hear Res 65: 118–124. 8458744 10.1016/0378-5955(93)90206-g

[22] Tremblay K, Kraus N, McGee T, Ponton C, Otis B (2001) Central auditory plasticity: changes in the N1–P2 complex after speech-sound training. Ear Hear 22: 79–90. 11324846 10.1097/00003446-200104000-00001

[23] Tremblay KL, Shahin AJ, Picton T, Ross B (2009) Auditory training alters the physiological detection of stimulus-specific cues in humans. Clin Neurophysiol 120: 128–135. 19028139 10.1016/j.clinph.2008.10.005 PMC2654261

[24] Zhang F, Hammer T, Banks HL, Benson C, Xiang J, et al (2011) Mismatch negativity and adaptation measures of the late auditory evoked potential in cochlear implant users. Hear Res 275: 17–29. 21129468 10.1016/j.heares.2010.11.007 PMC3061983

[25] Torppa R, Salo E, Makkonen T, Loimo H, Pykalainen J, et al. (2012) Cortical processing of musical sounds in children with Cochlear Implants. Clin Neurophysiol. 10.1016/j.clinph.2012.03.008 22554786

[26] Kelly AS, Purdy SC, Thorne PR (2005) Electrophysiological and speech perception measures of auditory processing in experienced adult cochlear implant users. Clin Neurophysiol 116: 1235–1246. 15978485 10.1016/j.clinph.2005.02.011

[27] Duncan CC, Barry RJ, Connolly JF, Fischer C, Michie PT, et al (2009) Event-related potentials in clinical research: guidelines for eliciting, recording, and quantifying mismatch negativity, P300, and N400. Clin Neurophysiol 120: 1883–1908. 19796989 10.1016/j.clinph.2009.07.045

[28] Naatanen R, Paavilainen P, Rinne T, Alho K (2007) The mismatch negativity (MMN) in basic research of central auditory processing: a review. Clin Neurophysiol 118: 2544–2590. 17931964 10.1016/j.clinph.2007.04.026

[29] Pakarinen S, Lovio R, Huotilainen M, Alku P, Naatanen R, et al (2009) Fast multi-feature paradigm for recording several mismatch negativities (MMNs) to phonetic and acoustic changes in speech sounds. Biol Psychol 82: 219–226. 19646504 10.1016/j.biopsycho.2009.07.008

[30] Naatanen R, Pakarinen S, Rinne T, Takegata R (2004) The mismatch negativity (MMN): towards the optimal paradigm. Clin Neurophysiol 115: 140–144. 14706481 10.1016/j.clinph.2003.04.001

[31] Stoody TM, Saoji AA, Atcherson SR (2011) Auditory mismatch negativity: detecting spectral contrasts in a modulated noise. Percept Mot Skills 113: 268–276. 21987925 10.2466/22.24.27.PMS.113.4.268-276

[32] Weise A, Bendixen A, Muller D, Schroger E (2012) Which kind of transition is important for sound representation? An event-related potential study. Brain Res 1464: 30–42. 22613810 10.1016/j.brainres.2012.04.046

[33] Aiken SJ, Picton TW (2008) Human cortical responses to the speech envelope. Ear Hear 29: 139–157. 18595182 10.1097/aud.0b013e31816453dc

[34] Naatanen R, Kujala T, Winkler I (2011) Auditory processing that leads to conscious perception: a unique window to central auditory processing opened by the mismatch negativity and related responses. Psychophysiology 48: 4–22. 20880261 10.1111/j.1469-8986.2010.01114.x

[35] Ollen JE (2006) A criterion-related validity test of selected indocators of music sophistication using expert ratings. Ohio: Ohio State University.

[36] Zeng FG (1994) Loudness growth in forward masking: relation to intensity discrimination. J Acoust Soc Am 96: 2127–2132. 7963026 10.1121/1.410154

[37] Klem GH, Luders HO, Jasper HH, Elger C (1999) The ten-twenty electrode system of the International Federation. The International Federation of Clinical Neurophysiology. Electroencephalogr Clin Neurophysiol Suppl 52: 3–6. 10590970

[38] Delorme A, Makeig S (2004) EEGLAB: an open source toolbox for analysis of single-trial EEG dynamics including independent component analysis. J Neurosci Methods 134: 9–21. 15102499 10.1016/j.jneumeth.2003.10.009

[39] Debener S, Hine J, Bleeck S, Eyles J (2008) Source localization of auditory evoked potentials after cochlear implantation. Psychophysiology 45: 20–24. 17910729 10.1111/j.1469-8986.2007.00610.x

[40] Viola FC, Thorne J, Edmonds B, Schneider T, Eichele T, et al (2009) Semi-automatic identification of independent components representing EEG artifact. Clin Neurophysiol 120: 868–877. 19345611 10.1016/j.clinph.2009.01.015

[41] Viola FC, Thorne JD, Bleeck S, Eyles J, Debener S (2011) Uncovering auditory evoked potentials from cochlear implant users with independent component analysis. Psychophysiology 48: 1470–1480. 21635266 10.1111/j.1469-8986.2011.01224.x

[42] Viola FC, De Vos M, Hine J, Sandmann P, Bleeck S, et al (2012) Semi-automatic attenuation of cochlear implant artifacts for the evaluation of late auditory evoked potentials. Hear Res 284: 6–15. 22234161 10.1016/j.heares.2011.12.010

[43] Terhaar J, Viola FC, Franz M, Berger S, Bar KJ, et al (2011) Differential processing of laser stimuli by Adelta and C fibres in major depression. Pain 152: 1796–1802. 21511396 10.1016/j.pain.2011.03.027

[44] Kiesel A, Miller J, Jolicoeur P, Brisson B (2008) Measurement of ERP latency differences: a comparison of single-participant and jackknife-based scoring methods. Psychophysiology 45: 250–274. 17995913 10.1111/j.1469-8986.2007.00618.x

[45] Caclin A, Brattico E, Tervaniemi M, Naatanen R, Morlet D, et al (2006) Separate neural processing of timbre dimensions in auditory sensory memory. J Cogn Neurosci 18: 1959–1972. 17129184 10.1162/jocn.2006.18.12.1959

[46] Makeig S, Westerfield M, Jung TP, Enghoff S, Townsend J, et al (2002) Dynamic brain sources of visual evoked responses. Science 295: 690–694. 11809976 10.1126/science.1066168

[47] Galvin JJ 3rd, Fu QJ, Oba S (2008) Effect of instrument timbre on melodic contour identification by cochlear implant users. J Acoust Soc Am 124: EL189–195. 19062785 10.1121/1.2961171 PMC2668986

[48] Gfeller K, Knutson JF, Woodworth G, Witt S, DeBus B (1998) Timbral recognition and appraisal by adult cochlear implant users and normal-hearing adults. J Am Acad Audiol 9: 1–19. 9493937

[49] Kong YY, Cruz R, Jones JA, Zeng FG (2004) Music perception with temporal cues in acoustic and electric hearing. Ear Hear 25: 173–185. 15064662 10.1097/01.aud.0000120365.97792.2f

[50] Billings CJ, Tremblay KL, Souza PE, Binns MA (2007) Effects of hearing aid amplification and stimulus intensity on cortical auditory evoked potentials. Audiol Neurootol 12: 234–246. 17389790 10.1159/000101331

[51] Naatanen R, Picton T (1987) The N1 wave of the human electric and magnetic response to sound: a review and an analysis of the component structure. Psychophysiology 24: 375–425. 3615753 10.1111/j.1469-8986.1987.tb00311.x

[52] Gfeller K, Christ A, Knutson JF, Witt S, Murray KT, et al (2000) Musical backgrounds, listening habits, and aesthetic enjoyment of adult cochlear implant recipients. J Am Acad Audiol 11: 390–406. 10976500

[53] Gfeller K, Witt S, Adamek M, Mehr M, Rogers J, et al (2002) Effects of training on timbre recognition and appraisal by postlingually deafened cochlear implant recipients. J Am Acad Audiol 13: 132–145. 11936169

[54] Driscoll VD, Oleson J, Jiang D, Gfeller K (2009) Effects of training on recognition of musical instruments presented through cochlear implant simulations. J Am Acad Audiol 20: 71–82. 19927684 10.3766/jaaa.20.1.7 PMC2784659

[55] Shahin A, Bosnyak DJ, Trainor LJ, Roberts LE (2003) Enhancement of neuroplastic P2 and N1c auditory evoked potentials in musicians. J Neurosci 23: 5545–5552. 12843255 10.1523/JNEUROSCI.23-13-05545.2003 PMC6741225

[56] Naatanen R, Kujala T, Escera C, Baldeweg T, Kreegipuu K, et al. (2011) The mismatch negativity (MMN) - A unique window to disturbed central auditory processing in ageing and different clinical conditions. Clin Neurophysiol. 10.1016/j.clinph.2011.09.020 22169062

[57] Schroger E, Paavilainen P, Naatanen R (1994) Mismatch negativity to changes in a continuous tone with regularly varying frequencies. Electroencephalogr Clin Neurophysiol 92: 140–147. 7511511 10.1016/0168-5597(94)90054-x

[58] Bishop DV, Hardiman MJ (2010) Measurement of mismatch negativity in individuals: a study using single-trial analysis. Psychophysiology 47: 697–705. 20210877 10.1111/j.1469-8986.2009.00970.x PMC2904495

[59] Naatanen R, Teder W, Alho K, Lavikainen J (1992) Auditory attention and selective input modulation: a topographical ERP study. Neuroreport 3: 493–496. 1391755 10.1097/00001756-199206000-00009

[60] Winkler I, Paavilainen P, Alho K, Reinikainen K, Sams M, et al (1990) The effect of small variation of the frequent auditory stimulus on the event-related brain potential to the infrequent stimulus. Psychophysiology 27: 228–235. 2247552 10.1111/j.1469-8986.1990.tb00374.x

