# Supplementary figures and images for: Temporal Feature Perception in Cochlear Implant Users
Source: PLoS One. 2012 Sep 21;7(9):e45375. doi: 10.1371/journal.pone.0045375 (PMC3448664; doi:10.1371/journal.pone.0045375)

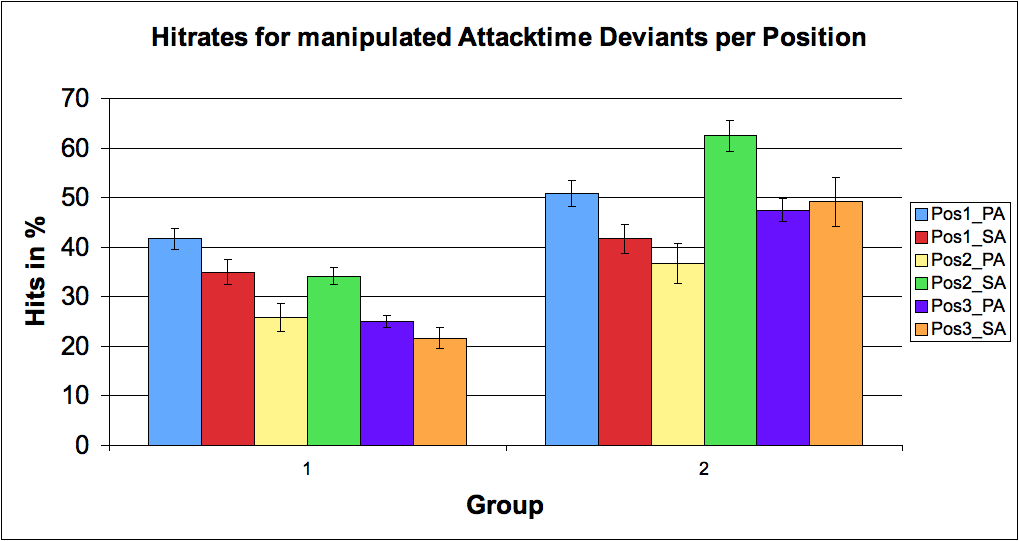

Supplement: Figure S1 — Hitrates for manipulated attack time Deviants per Position. Hitrates for different attack time manipulations in dependency on their position for CI users (Group1) and NH controls (Group 2). Asterisks indicate level of significance *p<0.05; **p<0.001. (TIF) [file pone.0045375.s001.tif]
