# Supplementary material for: Temporal Feature Perception in Cochlear Implant Users
Source: PLoS One. 2012 Sep 21;7(9):e45375. doi: 10.1371/journal.pone.0045375 (PMC3448664; doi:10.1371/journal.pone.0045375)
Supplement: Table S1 — Demography of the behavioral discrimination task participants. (DOC) [file pone.0045375.s002.doc]

Table S1

| Subject | Sex | Age  (years) | Implanted side | Implant type | Dur. of deafness (years) | Dur. of Implant use in month | Speechscores  Freiburger monosyllabic words test in quiet (%) |
| --- | --- | --- | --- | --- | --- | --- | --- |
| P1 | m | 20 | right | Nucleus | 14.42 | 56.04 | 85 |
| P2 | f | 20 | right | Medel Sonata | 3.1 | 9.96 | 85 |
| P3 | f | 56 | left | Nucleus | 40.92 | 11.64 | 65 |
| P4 | m | 39 | right | AB Clarion | 10.08 | 167.04 | 95 |
| P5 | m | 40 | right | Nucleus | 1 | 78 | 95 |
| P6 | m | 40 | left | AB Hires | 10.67 | 87.96 | 70 |
| P7 | f | 47 | left | Medel Pulsalr | 31.08 | 50.04 | 95 |
| P8 | m | 44 | left | Nucleus | 3.33 | 11.04 | 50 |
| P9 | f | 41 | left | Medel Sonata | 15.42 | 18.96 | 25 |
| P10 | m | 45 | left | Nucleus | 6.42 | 6 | 70 |
| P11 | m | 22 | left | AB CLarion | 0.1 | 115.08 | 90 |
| P12 | f | 43 | right | AB Clarion | 1.34 | 168 | 85 |
